# Supplementary material for: Enhanced Ferroelectric and Dielectric Properties of Niobium-Doped Lead-Free Piezoceramics
Source: Materials (Basel). 2023 Jan 4;16(2):477. doi: 10.3390/ma16020477 (PMC9865156; doi:10.3390/ma16020477)
Supplement: Supplementary file 1 [file materials-16-00477-s001.zip › materials-1886587-supplementary.pdf]

Supplementary data

FTIR:

In FTIR analysis, the O-H stretching modes are found at 3422 cm^{-1} (Figure S1). These bands are usually related to the absorbed water by KBr pellets used for FTIR analysis. The bands at 1630, 1427, 850, and 580 cm^{-1} correspond to the bismuth carbonate. The absorption peak found at 1427 cm^{-1} is related to the vibration modes of C=O [22]. This peak of C=O is observed possibly because of small traces of carbonates left in the mixture. A split in peak observed at around 1000 cm^{-1} after Nb additions (Figure S1) suggests the distortion caused at O-Ti-O by the replacement of Ti^{+4} by Nb^{+5} [22-24].

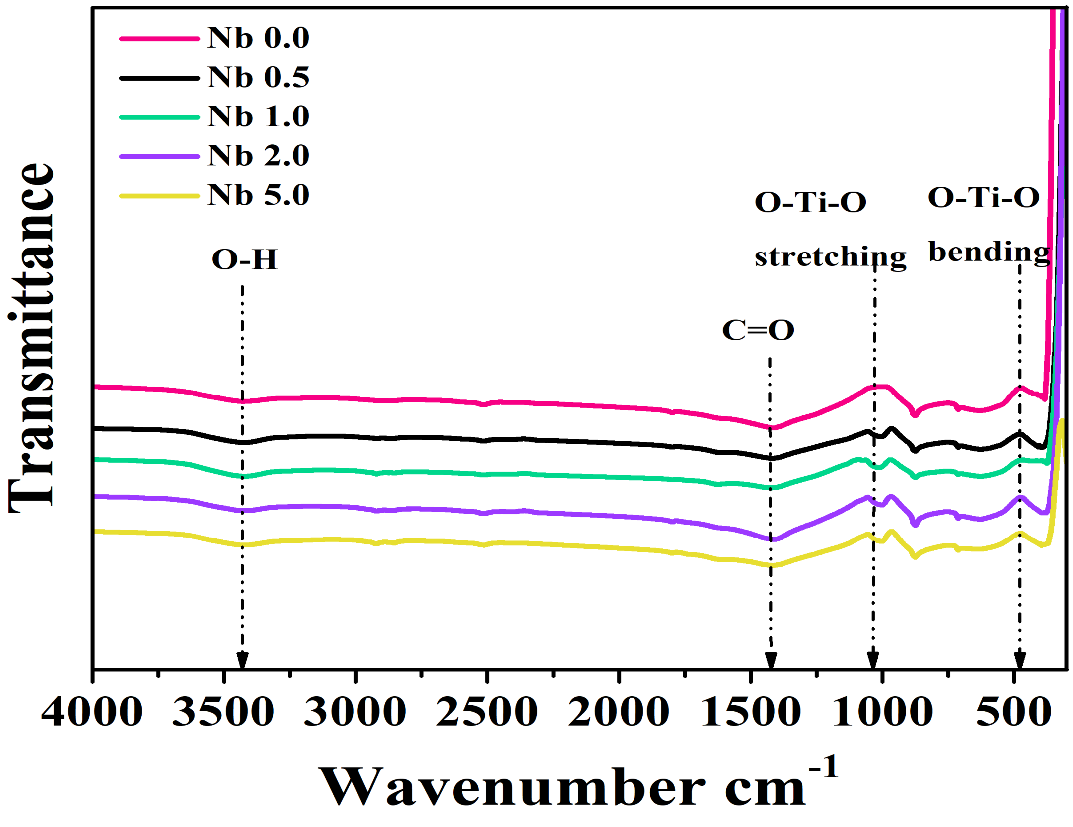

Figure S1 FTIR spectra after sintering of non-doped and Nb-doped BNT-ST26

Dielectric constant as a function of frequency:

Dielectric permittivity was calculated across the frequency of 100Hz to 5MHz shown in Figure S2. It was observed that the increase in frequency decreases the dielectric constant of all the materials with the increase in Nb addition. This trend of the curve can be described by the Maxwell-Wagner model. Maxwell-Wagner effect accounts for charge accumulation at the interface of conductive and nonconductive materials. The interface is working to prevent the flow of charge from conducting part to the non-conductive part and thus accumulates charges in proportion to the applied voltage. After the application of an electric field across the dielectric material, electrons start arriving at the grain boundaries by the mechanism of hopping. The grain boundaries are resistive [28]. This resistive nature results in charge accumulation and produces space charge polarization. The accumulated charge carriers require a little time to align themselves with the external field. At low- frequencies, this process is easier to maintain and hence high dielectric permittivity value at lower frequencies. The increase in frequency makes it difficult for the charge carriers to reach the grain boundaries and align with the external field causing a reduction in polarization and dielectric permittivity. [28,29] After the niobium substitution, the initial increase in the dielectric constant was observed compared to the non-doped BNT-ST peaking at concentration Nb 0.5 wt% but it decreases considerably as the concentration of Nb is increased in BNT-ST. The volatile Na and Bi content in BNT-ST causes point defects in the structure. These defects further contribute to the Maxwell-Wagner effect resulting in high dielectric permittivity and loss values in low-frequency range. Loss in dielectric permittivity at Nb concentration might be due to the increase in distortion caused by the replacement of Ti^{4+} ions by bigger Nb^{+5} ions at B-sites which results in defects like vacancies, mobile ions, or leaky grain boundaries which hinder the polarization [18,28-31].

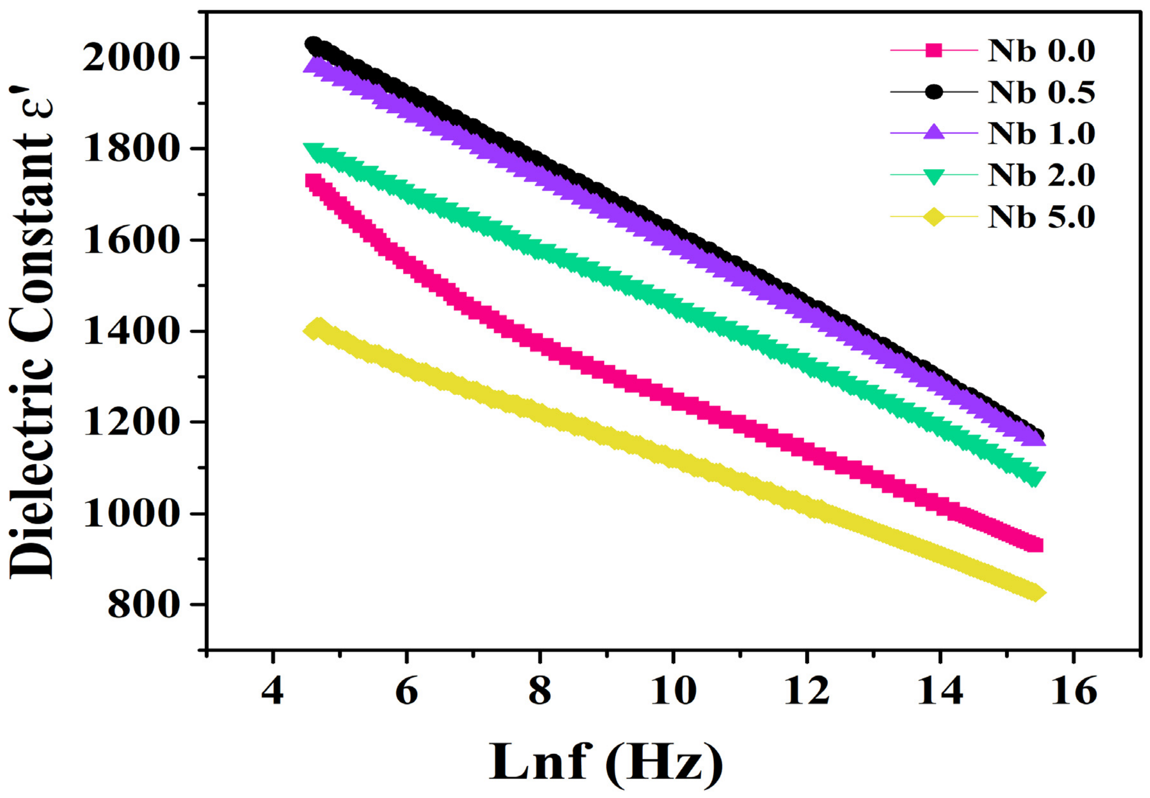

Figure S2 Dielectric constant non-doped and Nb-doped BNT-ST26

Dielectric loss is linked with the amount of energy dissipated. Dielectric loss is theoretically the imaginary part as the function of frequency (Figure S3). The increasing frequency increased the dielectric loss significantly for all samples. The increasing Nb concentrations in the BNT-ST26 decreased the dielectric loss in comparison to the undoped BNT-ST26 ceramic (Figure S3). This behavior can be understood by the role of grain boundaries as explained by Koop's theory [19]. Grain boundaries at lower frequencies play a more dynamic role because of their resistive nature. As a result, the mechanism of polarization requires more energy causing high values of dielectric loss [19,20].

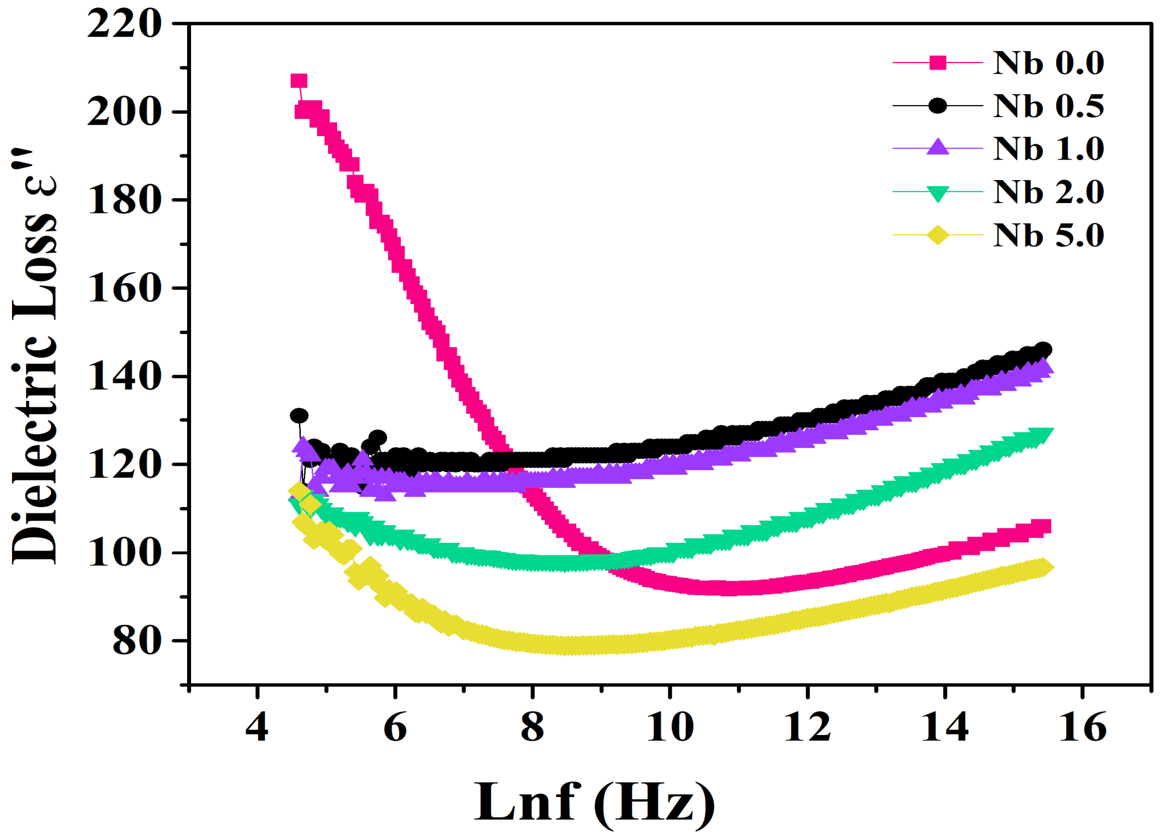

Figure S3 Dielectric loss of pure and Nb-doped BNT-ST26

Dielectric Tangent Loss:

The Dielectric tangent loss as a function of frequency is shown in Figure S4. The increase in dielectric tangent loss with the frequency is observed for all compositions (Figure S4). The tangent loss D is given by the proportion of dielectric loss and that of dielectric permittivity. The increase in the Nb concentrations up to 2% decreases the tangent loss and a then slight increase in this loss is observed for 5% Nb-doped ceramic at lower frequencies (Figure S4). This might be explained by the resistance provided by the active grain boundaries to the charge carriers which now need extra energy for polarizing. The dielectric tangent loss may depend upon various factors like stoichiometry, structure homogeneity, etc.

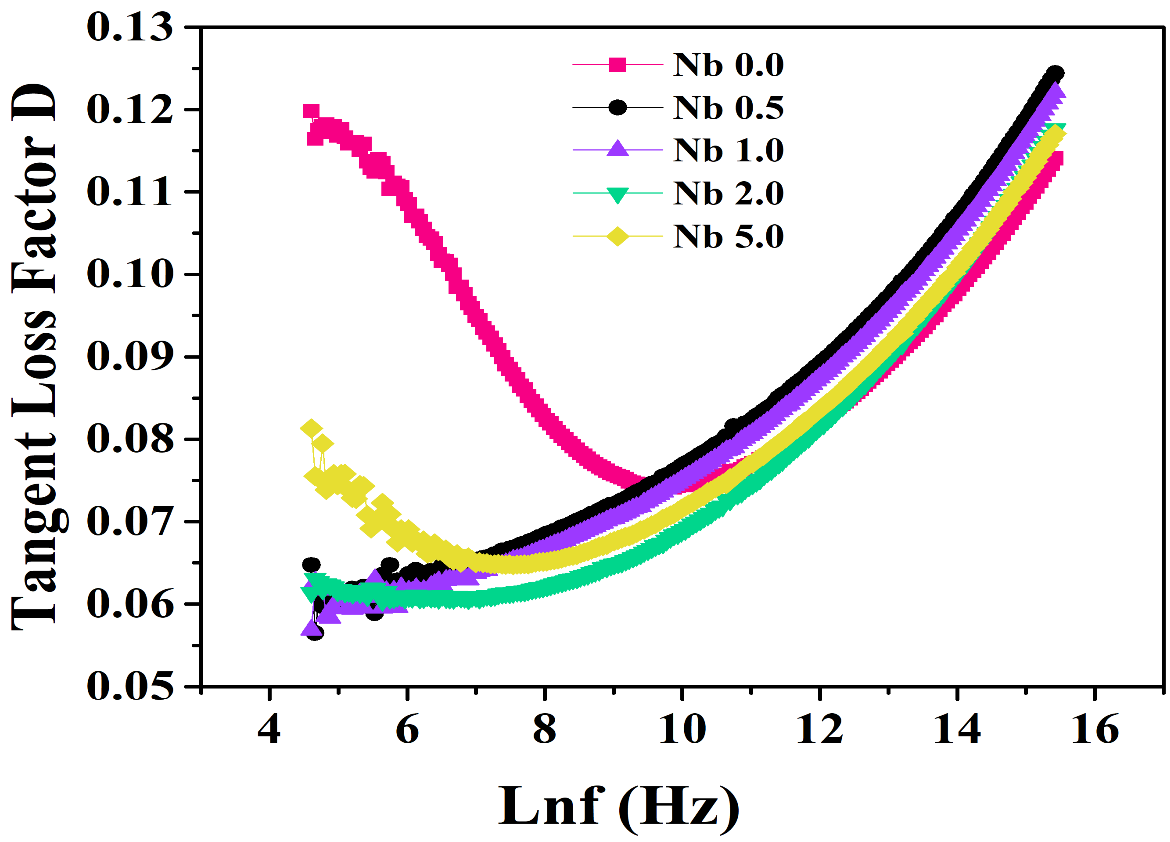

Figure S4 Dielectric tangent loss of non-doped and Nb-doped BNT-ST26

AC Conductivity:

The AC conductivity vs frequency was calculated in the frequency range of 100Hz-5MHz using the following relation;

$$\sigma_{ac} = \omega\, \varepsilon_o\, \varepsilon'\, \tan\delta$$

where ω is the angular frequency which is equal to 2πf (here f is frequency)

ε_o is known as the permittivity of free space,

Tanδ is the dielectric tangent loss factor and

ε' is the dielectric permittivity.

AC conductivity vs frequency curves is given in Figure S5. A significant increase in the AC conductivity was observed with increasing frequency for doped and non-doped compositions (Figure S5). The trend can be understood in terms of grain boundaries explained by Max-well and Wagner. The grain boundary is resistive and plays an effective part at lower frequencies

providing resistance to hopping of electrons, but at high frequencies, the conducting grains are more actively promoting the hopping mechanism of electrons AC conductivity of Nb 0.5 wt% was observed to be the highest. The conductivity increases because dopant has a conductive nature and it contributes to the increase in conductivity.

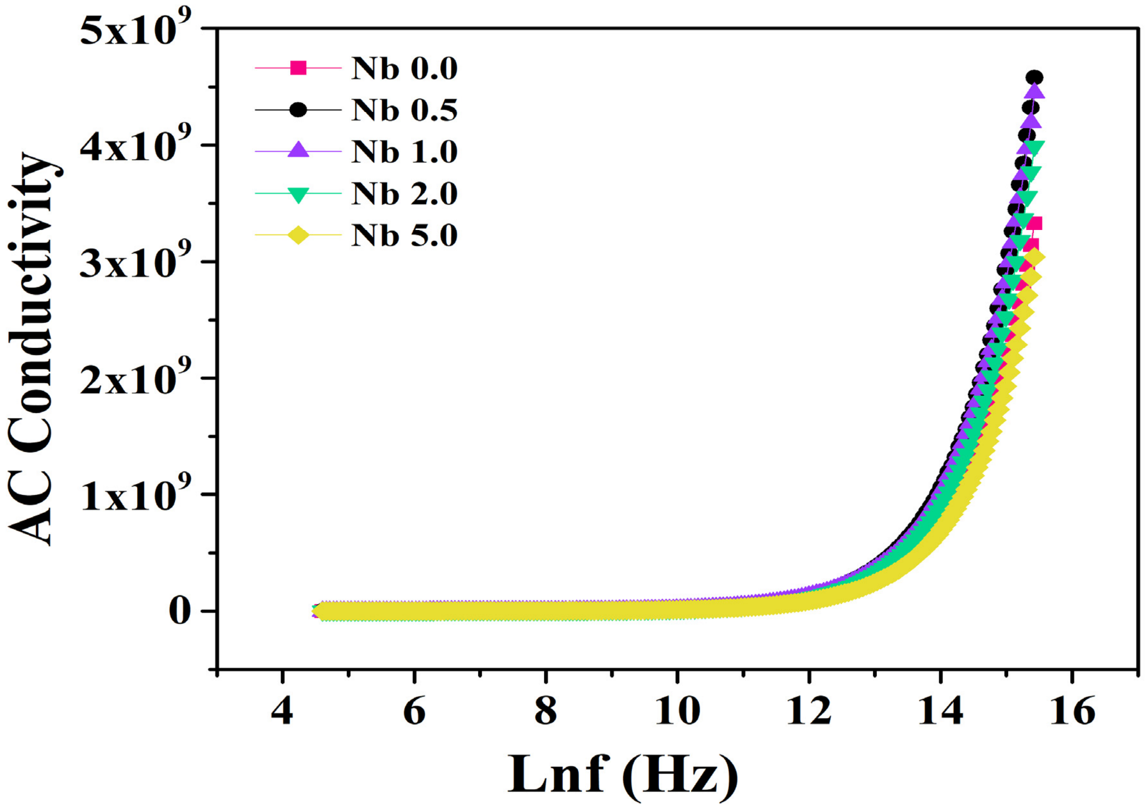

Figure S5 AC conductivity of pure and Nb-doped BNT-ST26

Impedance:

The impedance and frequency curve of BNT-ST-Nb ceramics are shown in Figure S6. Impedance is defined as the total resistance of the system. It was observed that the impedance decreased with the addition of niobium with a minimum value observed for 0.5wt%Nb doped BNT-ST26. This aging may be because of the conductive nature of the dopant.

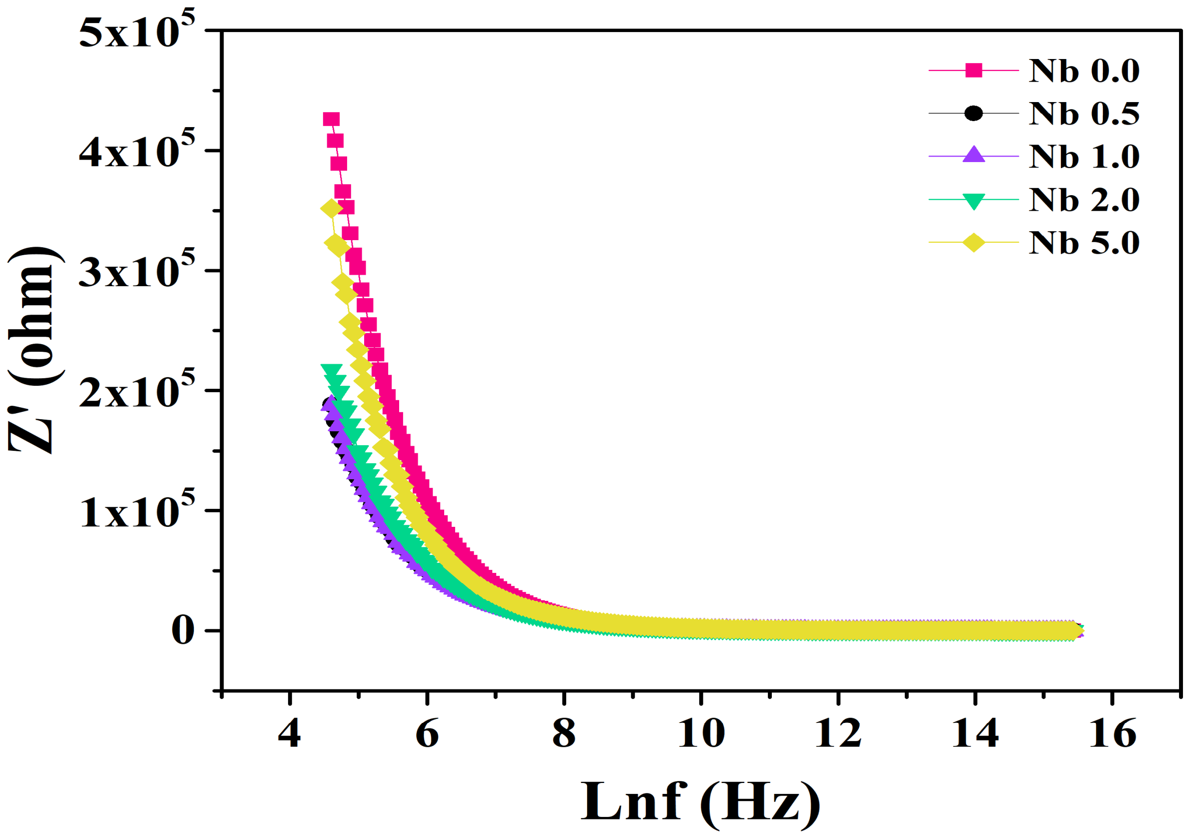

Figure S6 Impedance of non-doped and Nb-doped BNT-ST26.

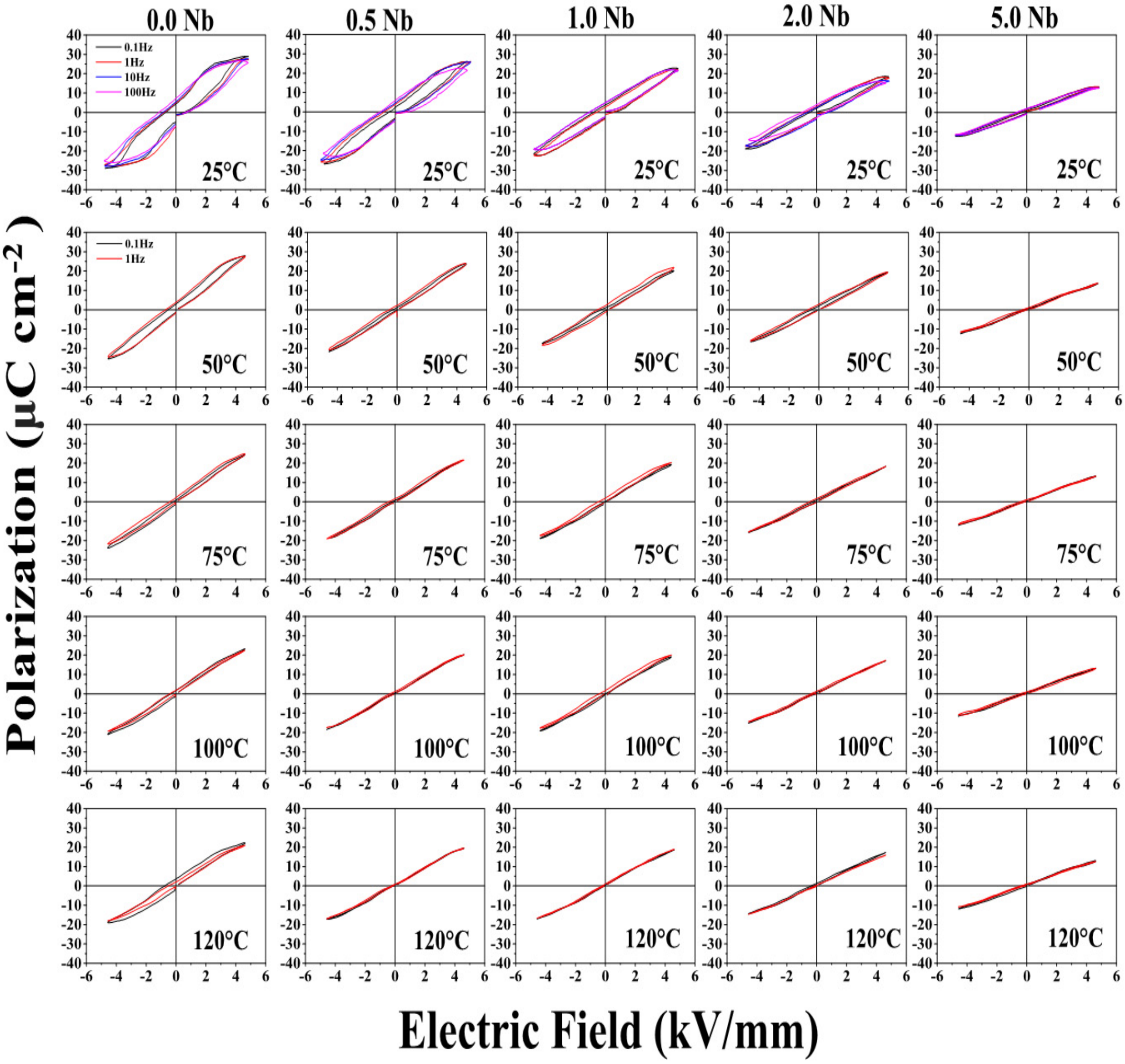

Figure S7 Polarization vs electric field graphs of pure and Nb-doped BNT-ST26 ceramic at a temperature range from room temperature to 120 ºC
